# On the Enjoyment of Sad Music: Pleasurable Compassion Theory and the Role of Trait Empathy

**DOI:** 10.3389/fpsyg.2020.01060

**Published:** 2020-05-28

**Authors:** David Huron, Jonna K. Vuoskoski

**Affiliations:** ^1^Center for Cognitive and Brain Sciences & School of Music, The Ohio State University, Columbus, OH, United States; ^2^RITMO Centre for Interdisciplinary Studies in Rhythm, Time and Motion, University of Oslo, Oslo, Norway; ^3^Department of Musicology, University of Oslo, Oslo, Norway; ^4^Department of Psychology, University of Oslo, Oslo, Norway

**Keywords:** music and emotion, sadness, trait empathy, compassion, fiction

## Abstract

Drawing on recent empirical studies on the enjoyment of nominally sad music, a general theory of the pleasure of tragic or sad portrayals is presented. Not all listeners enjoy sad music. Multiple studies indicate that those individuals who enjoy sad music exhibit a particular pattern of empathic traits. These individuals score high on *empathic concern* (compassion) and high on imaginative absorption (*fantasy*), with only nominal *personal distress* (commiseration). Empirical studies are reviewed implicating compassion as a positively valenced affect. Accordingly, individuals who most enjoy sad musical portrayals experience a pleasurable prosocial affect (compassion), amplified by empathetic engagement (fantasy), while experiencing only nominal levels of unpleasant emotional contagion (commiseration). It is suggested that this pattern of trait empathy may apply more broadly, accounting for many other situations where spectators experience pleasure when exposed to tragic representations or portrayals.

## Introduction

Over 2000 years ago, Aristotle drew attention to an apparent paradox concerning the enjoyment of artistic portrayals related to tragic events. In his *Poetics*, Aristotle wondered how a spectator or audience member might come to enjoy representations of negatively valenced events, notably events that evoke pity or fear. The so-called “problem of negative emotions in the arts” has been the topic of considerable theorizing among aesthetic philosophers. Classic literature pertaining to the topic includes writings by Dubos, Hume, Diderot, Kant, Burke, Schopenhauer, and Nietzsche, among others. More recently, a new generation of philosophers has expanded the debate (e.g., see the edited compilation by Levinson, 2014). Talon-Hugon (2014) has conveniently characterized the paradox via the following informal syllogism:

(1) Negative emotions are psychologically unpleasant.
(2) Art can cause us to experience negative emotions.
(3) We find pleasure in this experience.

Clearly, at least one of the three statements in this syllogism must be wrong. Philosophers have proposed a number of possible solutions. Aristotle himself emphasized the positive value of imitation in artistic portrayals. Dubos (1719/1733) and Hume (1757/1993) drew attention to the utility of negative emotions in dispelling boredom. Many theories stress the importance of the “make-believe” or fictive character of arts and entertainments (e.g., Walton, 1990; Schubert, 2016), or that spectators benefit by the feeling of control that arises from our ability to terminate the artistic experience at will (e.g., Morreall, 1985). Another common approach draws on the pleasure of learning from the misfortunes of others, or the appeal of informative gossip (e.g., Robinson, 1995; De Clercq, 2014). Others point to the antecedent feelings that motivate information gathering, namely the positive experience of curiosity (e.g., Korsmeyer, 2014). Yet other theorists argue that pleasure is not the only artistic value (Levinson, 1996: 18), or that unpleasant experiences can be intrinsically worthy (Smuts, 2007, 2009). With only a few exceptions (e.g., Juslin, 2013; Schubert, 2016; Menninghaus et al., 2017) most theories fail to engage the pertinent empirical literature. Relevant literature includes notable physiological, psychological, and ethological studies – especially extant research related to empathy and fear.

Many of the existing theories hinge on the value of narratives that convey potentially useful information. For such accounts, instrumental music raises notable challenges. Compared with story-telling, it is hard to identify what life lessons might be learned from listening to instrumental music; music does not obvious satisfy our curiosity for human gossip or inspire preventive thoughts about how to escape a murderous villain. Also, musical narratives are more obviously fictive: the artifice is overt and it is rare that a musical work might warrant the claim “based on a true story.” Although instrumental music can sometimes illustrate concrete scenarios – like the scene-by-scene programmatic depiction of Wellington’s 1813 victory over Joseph Bonaparte in Beethoven’s Opus 91—musical “narratives” are rarely conveyed in anything but the broadest of brush strokes.

An additional difference between music and narrative stories can be found in the concept of agency. Although scholars have rightly drawn attention to how instrumental music exhibits animacy cues suggestive of conscious agency (e.g., Broze, 2013; Hatten, 2018), the evoking of character or persona in instrumental music is clearly more problematic than in literature, drama, film, much pictorial art, or dance (Davies, 1997a).

In short, although theoretical accounts that attempt to resolve the paradox of negative emotions by appealing to narrative properties may capture some aspects of the enjoyment of sad or tragic arts, they are unlikely to apply to putative sad or tragic instrumental music. That is, nominally sad music offers a particularly challenging test case for resolving the apparent paradox of the enjoyment of negative emotion in art and entertainment.

Several theoretical accounts posit some sort of process of “transmutation” by which a negatively valenced affect (such as sadness) is transformed into something positive. Eerola et al. (2017) have drawn attention to the inherent difficulty of such accounts. Transformative accounts typically beg the question of timing: at what point in time does a negative affect become transmuted into a positive feeling state? In theatrical drama, for example, must an audience member wait until the defeat of the villain before experiencing a positive emotion? If so, how can we explain the potential for enjoyment prior to the denouement? In the present account, we propose a theory that circumvents the problem of transformation by positing a positive emotion that is likely to be evoked throughout a tragic scenario or portrayal. That is, we will point to evidence challenging the second statement in Talon-Hugon’s syllogism. In our account, we will emphasize the role of empathy, and in particular, the experience of compassion. The empirical work on which our account is based pertains almost exclusively to the apparent paradox of the enjoyment of nominally sad music. However, we will claim that our proposed resolution of this paradox is likely to apply more broadly to other tragic arts and entertainments.

## Individual Differences

It bears highlighting that not all arts and entertainment experiences are enjoyable. Not everyone enjoys watching horror films or coping with emotionally overwrought dramas. People can find certain works disturbing, upsetting, offensive, or repellent. When viewing a tragedy, for example, a spectator might well be moved to true grief, marked by weeping and accompanied by distinctly unpleasant feelings. Indeed, people will leave a movie theater in mid showing, stop reading a book, or turn off the music when moved to the point of acute sadness or grief. Behaviors that terminate the experience suggest that a sad or tragic work is not enjoyable for the spectator.

In the specific case of music, surveys indicate that not everyone enjoys listening to nominally sad works or passages. In a survey of music students, Garrido and Schubert (2011) found that just half of the respondents (30 out of 59) agreed or strongly agreed with the statement “I like to listen to music which makes me feel sadness or grief.” Similarly, Taruffi and Koelsch (2014) found that 54% of their 772 respondents gave a rating of 5 or higher on a 7-point Likert scale (1 = strongly disagree, 7 = strongly agree) for the statement “When I am in a sad mood I like to listen to sad music.” However, only 33% gave a rating of 5 or higher for the statement “When I am in a positive mood I like to listen to sad music” (Supplementary Material; Taruffi and Koelsch, 2014).

In an effort to poll a sample less dominated by people of European descent, we conducted individual interviews over a 3-year period with 35 taxi drivers. Although biased toward males, interviewees included drivers from China, Egypt, Ethiopia, Greece, Haiti, Kashmir, Mali, Nigeria, Pakistan, Punjab, Russia, Serbia, Somalia, Tunisia, and Vietnam. Interviews included the following question: “Some people like listening to sad music; and some people don’t like listening to sad music. Which are you?” Of the 31 interviewees who provided unambiguous answers, 18 (58 percent) reported that they like listening to nominally sad music with 13 (42 percent) reporting that they do not like sad music.

It should be noted that none of these three surveys distinguished instrumental from vocal music with lyrics. Nor did any of the studies control for familiarity or for possible learned autobiographical associations. Nevertheless, such studies appear to highlight a wide range of individual responses. This suggests that any purported explanation regarding the enjoyment of sad music should include some experiential and/or dispositional factor that might account for the observed individual differences.

Apart from the enjoyment of nominally sad music, a further debate centers on the question of whether music can evoke genuine sadness in listeners. Garrido and Schubert (2011) chronicle some of the many voices in this ongoing debate, identifying roughly equivalent numbers of scholars proposing that music can, or cannot, evoke true sadness. Garrido and Schubert go on to note that the theoretical views of different scholars appear to reflect the individual personal experiences of the author proposing the theory. Indeed, several authors explicitly state that their theory was motivated by reading reports by others that don’t resonant with their own experience. In light of the many appeals to personal experience, Garrido and Schubert suggest that this controversy amounts to further evidence of wide individual differences; these differences parallel the wide variance found in surveys probing the enjoyment of nominally sad music.

## Understanding Emotional Displays

Our proposed theory builds on an ethological understanding of emotional displays. Specifically, ethological signaling theory offers a somewhat different perspective on emotional displays than is common in psychology. Before presenting our theory, it is appropriate to review some basic ethological concepts, and identify how this approach differs from commonly held views.

Much research on emotion commonly presupposes what might be called the “emotional communication model.” This model assumes that the purpose of an emotional display is to communicate one’s emotion to potential observers. The model might be summarized by the following scenario:

I feel happy.
This causes me to smile.
You observe my smile.
And you infer that I must feel happy.
(This may or may not cause you to feel happy as well).

Although this scenario is widely presumed in many discussions of emotion, it is problematic from an ethological and evolutionary perspective (Huron, 2015a, b, 2018). An ethologist would ask the question: what is to be gained by broadcasting one’s emotional state to a general environment? As in the game of poker, an honest communication of one’s emotional state merely provides information for others to use for their benefit, not necessarily for the benefit of the displayer.

In ethological theory, the purpose of a display (formally, a signal) is to change the behavior of an observer to the mutual benefit of the displayer and observer (Maynard Smith and Harper, 2003). If a display proves detrimental to the displayer, selection pressure would ultimately encourage the elimination of the display. That is, displays should arise, only if the behavior typically enhances the inclusive fitness of the displayer. Indeed, although some emotions (such as disgust, anger, and grief) are associated with distinctive displays, most affective states (loneliness, affection, anxiety, suspicion, nostalgia, regret, pity, pride, etc.) exhibit no characteristic display. Even basic feelings of hunger or thirst are clandestine feeling states. According to signaling theory, the incentive for generating a display (such as smiling or making an angry facial expression) is to change the behavior of the observer so as to benefit the individual making the display. At the same time, in order to ensure a predictable or stereotypic response by the observer, some benefit must also typically accrue to the observer.

From an ethological perspective, the purpose of a smile (whether voluntary or involuntary) is not to communicate that one is happy. Indeed, people commonly smile when experiencing stress or as an act of deference in the presence of a person of high social status. People laugh when amused, but people commonly giggle or laugh in the face of fear, and will laugh at others as a form of mocking aggression. Similarly, the purpose of weeping is not to communicate that one is sad. Indeed, people commonly weep when feeling joy, laughter, pain, patriotism, and other emotions. Famously, female fans of *The Beatles* wept in the presence of their idolized and adored stars.

As observers, without the benefit of context, we do not know whether a smile arises due to happiness or stress, whether laughter arises from amusement, fear, or aggression, or whether tears arise from grief, pain, celebration, patriotism, or adoration. Of course, if a person has just lost his job, we might infer that the tears arise from grief; when a child falls off her bicycle, we might infer that the tears arise from pain; when a beauty pageant contestant wins, we might infer that the tears arise from joy. By themselves, smiling, laughter, and weeping do not convey the emotional state of the signaler. Instead, as observers, we do our best to decipher the likely emotion motivating the display by attending to context. Of course, much smiling does indeed arise from happiness and much weeping arises from grief. But that doesn’t mean that a smile means happiness, or weeping means grief.

So what is the purpose of an emotional display if not to communicate a person’s emotion? In a classic paper from 1978, Dawkins and Krebs convincingly argued that the purpose of displays is manipulation, not information. Signals exist, not to inform observers of the emotional state of the signaler, but to manipulate the behavior of the observer.

So what is the purpose of sadness-related displays like weeping, if not to communicate sadness? In the case of weeping, Kottler and Montgomery (2001) have cogently argued that the purpose of weeping is to encourage observers to terminate aggression and offer altruistic assistance to the weeping individual. Sadness displays motivate such changes of behavior by evoking feelings of prosocial compassion in observers.

Consider the case of the weeping beauty pageant winner. Why would someone who is likely experiencing “joy” weep? The effect of the weeping is best understood if we contrast weeping with gloating celebration. Suppose the response of a beauty-pageant winner was akin to the triumphant boasting of a boxing champion. Turning to the other contestants, she thrusts her arms into the air and declares “I am the greatest” or “Tough luck suckers!” In the case of beauty pageants, personality counts for something. We prefer winners to be magnanimous, generous, self-effacing, agreeable – not self-centered or egotistical. The effect of weeping is to evoke prosocial feelings in the audience. We become warmly disposed toward her, perhaps ready to offer her assistance if necessary. But the motivation for her weeping is not grief; instead, the weeping represents a powerful declaration of humility. As observers, we interpret the display as the polar opposite of gloating, namely, modesty.

So what is the commonality that unites all forms of weeping? The commonality is to be found in the evoked feelings of the observers, not that of the weeping signalers. In all of the various cases where people weep, the weeping tends to evoke a broadly prosocial disposition in observers. This prosocial attitude may manifest itself in the termination of aggression, compliance to the explicit requests or desires of the weeper, the offering of altruistic assistance even in the absence of overt requests, a feeling or connection or bonding, or simply a favorable attitude toward the weeping individual.

In both the case of the grief-stricken weeper and the weeping beauty-pageant winner, the result is to evoke positive prosocial feelings in observers. Paradoxically, signal displays are more predictive of the induced emotional states of observers than the emotional states of displayers. In understanding emotional displays, emotion researchers have been generally looking at the wrong person.

What ethological theory tells us is that the principal purpose of nominally sad expressions like weeping is to evoke prosocial compassion in observers, not to communicate a presumed saddened state of the displayer. In short, the evoking of prosocial compassion is not simply some incidental artifact of observing sadness, but the main goal.

It should be added that this ethologically inspired way of understanding displays applies only to a small subset of display behaviors that meet stringent criteria for the designation *signal*. Signals are innate behavioral interactions that have evolved because they benefit both the signaler and the observer (More about the benefits for observers later). The vast majority of animal displays are ethological *cues* that involve very different forms of interaction. Human behavior manifests only a small handful of displays that might qualify as ethological signals. Huron (2018) assembled a detailed case consistent with the claim that human weeping conforms to an ethological signal.

Apart from sadness expressions such as weeping, do we have any evidence that nominal sad music is able to act as an ethological signal? Apparently, yes. In the case of sad music, much evidence supports the concept of a voice-music homolog—where music emulates the acoustical features of melancholic or grief-related vocalizations (Juslin and Laukka, 2003; Schutz et al., 2008; Paul and Huron, 2010; Huron et al., 2014). Overall, the research suggests that music and vocal prosody share common affective resources for both production and perception. These shared mechanisms need not necessarily relate directly to shared acoustical features, but may arise from phylogenetically primitive mechanisms relating to movement or posture (Sievers et al., 2013). These shared aspects have led some researchers to suggest that even purely instrumental music has some capacity to act as a virtual person (e.g., Watt and Ash, 1998; Parncutt and Kessler, 2006).

If the purpose of sad displays is to evoke prosocial dispositions in listeners, why would many listeners claim that nominally sad music makes them feel sad?

## Contagious and Repercussive Emotions

In considering the emotional response of an observer to some display, it is appropriate to distinguish *contagious* emotions from *repercussive* emotions. Contagious emotions refer to those induced affects where the observer simply echoes the emotion associated with the displaying individual. By *repercussive* emotions, we mean an induced affect that typically arises in response to the emotional display but differs from that of the displayer. In the empathy literature this distinction is evident in the difference between true empathy (feeling the same emotion as a displayer) and sympathy (an emotion induced by the display, but which differs from the emotion motivating the displayer).

In the case of nominally sad music, the prime candidate for a contagious response is an induced feeling of *sadness* in the observer. The prime candidate for a repercussive response is an induced feeling of sympathy or *compassion*. For each of these responses we might address two questions: What is the mechanism that induces the response? And what is the purpose of the response itself?

A possible candidate mechanism for inducing contagious emotions can be found in mirror neurons. Rizzolatti, Gallese, and their colleagues have suggested that mirror neurons offer a plausible mechanism for general emotional contagion (Gallese, 2003; Bråten, 2007; Rizzolatti and Sinigaglia, 2008; Iacoboni, 2009). Molnar-Szakacs and Overy (2006) and Overy and Molnar-Szakacs (2009) have more explicitly proposed that mirror neurons are responsible for emotional contagion in the specific case of music.

Note that emotional contagion via mirror neurons may or may not serve any useful function. For example, Heyes (2010) has argued that mirror neurons may simply be non-functional byproducts of associative learning. More broadly, it may be that emotional contagion (whatever its source) is merely an artifact of some other brain process and by itself serves no adaptive purpose. Nevertheless, a number of speculative theories have been proposed concerning possible functions for emotional contagion (Hatfield et al., 1993). At least three benefits have been proposed. One potential benefit is that emotional contagion may enhance emotion recognition by an observer. That is, some vicarious feeling alerts the observer to the emotional state of the displayer. Even if the intensity of the evoked emotion is rather subdued, the vicarious mirroring of a displayed feeling may still prove useful for inferring someone’s emotional state.

In other circumstances, it may be beneficial for the observer to fully mirror the affective state of the displayer. Especially among close social partners or social groups, there may be considerable benefit for the emotion to be fully shared among the group members. For example, close romantic partners might benefit by sharing the sadness of one member. Specifically, sharing the same emotion enhances social bonding (for a review, see Seyfarth and Cheney, 2013).

In yet other circumstances, widespread emotional contagion might prove beneficial insofar as it facilitates coordinated group action. A group, such as a sports team, is likely to produce more effective joint action when all of the members of the group share the same emotion (Bryant, 2013).

In summary, plausible benefits of emotional contagion might include emotion recognition, social bonding or affiliation, and the facilitation of effective group action (Hatfield et al., 1993).

These possible benefits notwithstanding, unfettered emotional contagion would seem to have little general value. One might ask what benefit is your expression of anger if the sole result is that observers also become angry? The best response to many emotional displays is not merely to echo back the same emotion. Accordingly, it is appropriate to consider the role of repercussive emotions.

With regard to mechanism, the prime candidate for inducing repercussive emotions is a co-evolved ethological signal. In signaling theory, both strong empirical and strong theoretical grounds point to evolved responses that are innate, automatic, and largely resistant to executive control (Maynard Smith and Harper, 2003). Compared with other animals, humans exhibit a remarkable capacity for cognitive control by which emotional impulses can be impeded or suppressed. However, in the case of weeping, our disposition to weep in certain circumstances, and our disposition to respond when we observe someone weeping, are commonly resistant to executive control. For example, Gelstein et al. (2011) have assembled experimental evidence that psychic tears contain a pheromone that unconsciously reduces libido and aggression in males who are exposed to them. Huron (2018) has reviewed evidence documenting the limited executive control evident in both evoking weeping and in responding to a weeping individual.

With regard to function, here it is easy to imagine scenarios in which an appropriate emotional response deviates from the emotion being displayed. When observing an expression of anger or aggression, for example, it might be more appropriate for an observer to experience fear. When observing an expression of joy, in some circumstances it might be appropriate for an observer to experience envy. When observing the angst of a foe or enemy, one might expect an observer to experience some degree of delight or relish. And of course, when observing grief, it might be more appropriate for an observer to experience a feeling of pity or compassion rather than merely partake of the grief.

In the presence of an expression of sadness, one might expect that both contagious and repercussive responses can be induced. That is, emotional contagion would lead at least some observers to also experience some element of sadness, whereas a repercussive emotion might take the form of compassion or sympathy.

Since sadness is regarded as a negatively valenced emotion, we might suppose that the evoking of sadness in an observer would be experienced negatively. So what about compassion? Four key questions arise: (1) Do we have evidence that both sadness and compassion can be evoked simultaneously? (2) Do we have evidence that compassion is a positively valenced emotion? If so, (3) Are there circumstances where the positive repercussive feeling of compassion would dominate contagious feelings of sadness? And finally, (4) Do we have evidence that the proportion of positive and negative affects accounts for the observed individual differences in the enjoyment of nominally sad music? As we will see below, the empirical evidence suggests that the answer to all four questions is yes.

## The Pleasure of Compassion

Above we emphasized that the purpose of an ethological signal is to change the behavior of an observer to the benefit of the signaler. However, ethologists also agree that, in order for a signal to evolve, signals must benefit the observer as well (Maynard Smith and Harper, 2003). This suggests that we consider what benefit arises for an observer when a grief or sadness expression induces a feeling of compassion. Behaviorally, the feeling of compassion commonly leads to acts of altruism. Consequently, one might expect the benefits of compassion to be linked to the benefits of engaging in altruistic acts.

Evolutionary theory posits at least five benefits by which altruistic acts can be biologically adaptive by enhancing an individual’s inclusive fitness. First, selfless acts that assist closely related individuals can enhance inclusive fitness through kin selection (Haldane, 1932; Hamilton, 1963). Especially when the cost of assistance is low, assisting one’s closest relatives can have a marked facilitating effect in propagating shared genes. Trivers (1971) noted that helping people who are not kin can also benefit an individual through reciprocal altruism: the person you help today may feel obliged to help you in some future hour of need (see also Axelrod and Hamilton, 1981). Apart from the expectation of some sort of *quid pro quo*, a third benefit is an enhanced social status for the observer with respect to the stressed individual.

When our behaviors are conducted in the presence of an audience, or when gossip is able to communicate our behaviors to a wider group beyond immediate observers, a fourth mechanism comes to the fore: when we interact with the same people repeatedly over years or decades, it becomes adaptive for individuals to attend to reputation maintenance (Haley, 2002; Milinski et al., 2002; Haley and Fessler, 2005; Semmann et al., 2005; Silk and Boyd, 2010). A reputation for altruism and cooperation has notable long-term social benefits. Finally, a fifth mechanism adds punishment to the equation. Even a single defection or social violation can lead to long-term mistrust and suspicion. Failing to come to a person’s assistance can lead to social ostracism or social punishment (Silk and Boyd, 2010).

These five biological benefits constitute the distal or *ultimate* incentives encouraging altruistic behaviors. However, the *proximal* motivation for engaging in altruistic behaviors is not kin-selection or the pursuit of social status, but the feeling of *compassion*. Since altruistic behaviors evolved because of their ultimate benefits, we might expect the feeling of compassion to be positively valenced. That is, if altruistic acts are generally beneficial to the individual engaging in those acts, we should not be surprised that the motivating feelings would be experienced as positive. Our claim here is that compassion must be positively valenced, not negatively valenced. For convenience, we might refer to this account as *pleasurable compassion theory*.

What evidence do we have that compassion is indeed a positively valenced affect? In the first instance, anecdotes abound regarding the positive feelings associated with altruistic acts. Throughout history, many observers, including Saint Augustine, Thomas Hobbes, and Immanuel Kant argued that acts of charity are not truly altruistic—precisely because they make us feel good. When asked why he had given sixpence to a beggar, Thomas Hobbes famously noted “I was in pain to consider the miserable condition of the old man; and now my alms, giving some relief, doth also ease me.”

Apart from such anecdotal evidence, additional evidence of the pleasures of charity can be found in a handful of neuroimaging studies. In a study of charitable acts, Harbaugh et al. (2007) conducted brain scans while participants made various choices for the disbursal of a $100 account. They found that deciding to donate money to a local food bank was accompanied by activity in brain regions anatomically and functionally related to pleasure. For half of their participants, activation of the purported pleasure circuit was stronger when donating money than when receiving a monetary gift.

The Harbaugh et al. (2007) experiment was cleverly designed so that no one, apart from the participant, was aware of their decision whether or not to donate money. That is, the experiment controlled for the possibility that the motivation for charitable acts was the possibility of enhancing one’s social reputation. This raises the question of whether improving one’s reputation provides a further independent motive to engage in altruistic behaviors.

Empirical evidence can be found in an experiment by Izuma et al. (2008). While scanning participants’ brains, Izuma et al. (2008) gave controlled feedback from (fictitious) judges, who assessed the participant’s trustworthiness. The research results indicate that the social reward of a good reputation in the eyes of others results in activation of brain regions anatomically and functionally related to pleasure. In other words, acts of altruism are pleasurable in themselves, but further pleasure can arise if such acts are thought to result in an enhanced social reputation.

Notice that a person can feel compassion without acting altruistically. That is, one can feel pity or sympathy for someone, yet not be motivated to help. Moreover, if there is no observable altruistic behavior, then one’s social reputation cannot be enhanced. This raises a further question of whether the feeling of compassion is itself positively valenced in the absence of any altruistic act.

The authors are unaware of any research that directly addresses the question of whether the feeling of compassion itself is positively valenced. Nevertheless, extensive pertinent research exists regarding what might be called the “pleasures of opportunity.” An animal that is not hungry can still appreciate the discovery of a stash of food. A person may welcome a job offer, even if the person has no intention of switching employers. Someone may appreciate finding a lottery ticket, even though the ticket has virtually no value apart from the (highly unlikely) possibility of future gain. There are many examples of pleasures that are linked to opportunity rather than actual rewards.

A weeping individual offers the observer an opportunity to engage in altruistic or charitable acts which we know to result in positive feelings. The situation offers an option to enhance one’s reputation, to build social capital, or merely to feel virtuous. At face value, we might then expect compassion to be a positively valenced feeling, even if that feeling doesn’t necessarily lead to specific acts of altruism. Empirical evidence in support of this idea is to be found in classic research on dopamine. Research has long established how, over time, the release of dopamine shifts from consummatory behaviors to anticipatory behaviors (Weiss et al., 1993; Berridge and Robinson, 1998; Gebauer et al., 2012). That is dopamine rewards become more closely linked to “wanting” rather than “liking.” Initially, dopamine is released in response to the act of consumption: we enjoy the act of eating some previously unencountered food, for example. With experience, however, the release of dopamine peaks in anticipation of the reward. That is, there are strong feelings of pleasure that precede (and so motivate) an expected consummatory phase—whether or not consumption actual occurs.

In light of the study by Harbaugh et al. (2007) showing that altruistic acts activate the medial forebrain pleasure circuit, we would expect that the mere prospect or opportunity to engage in altruistic acts would itself be positively valenced. In fact, given the extensive existing research related to dopamine, it would be unusual to discover that the opportunity to behave altruistically is not experienced as pleasurable. In short, although no imaging study has yet tested whether brain systems associated with pleasure are activated in response to feeling compassion (in the absence of altruistic acts or thoughts), there are nevertheless good reasons to suppose that compassion, by itself, is experienced as a positively valenced affective state.

## Mixed Emotions

To this point, we have highlighted two possible emotional responses when someone observes a stressed individual – contagious and repercussive responses. As we have seen, signaling theory suggests that the purpose of a sadness display like weeping is to induce a feeling of compassion. At the same time, the phenomenon of emotional contagion suggests that displays of nominal sadness might be expected to induce parallel feelings of sadness in observers. This raises the question of whether it is possible to experience two affective states simultaneously. What evidence do we have that such presumed “mixed emotions” are possible?

Considerable evidence suggests that mixed emotions are commonplace (Schimmack, 2001; Larsen and McGraw, 2011). A meta-analysis by Berrios et al. (2015) examined 63 studies and concluded that mixed emotions are “a robust, measurable and non-artifactual experience.” If we accept that mixed emotions are possible, a separate question is whether mixed emotions are possible when the valences are diametrically opposed? That is, is it possible to experience positive and negative affects simultaneously? In the case of film scenes, Larsen and Green (2013) assembled evidence consistent with the inducing of concurrent positive and negative mixed emotions characteristic of a bittersweet experience. In the case of music, studies by Hunter et al. (2008, 2010) and Larsen and Stastny (2011) provide experimental evidence of concurrent happy and sad induced emotions.

Although an observer of grief may experience mixed positive and negative feelings, it is not necessarily the case that both feelings are equally salient or noticeable. In particular, when interacting with a sad or grieving individual, we may not be cognizant of the positive feelings associated with compassion. When we witness someone weeping, for example, our experience is not necessarily one of glee or elation at the prospect of being able to help. Suppose your beloved pet had died and you are crying in the presence of a friend. Suppose further that your friend relayed the following sentiment to you: “Thanks so much for giving me the pleasurable opportunity to console you in your moment of need.” Such an expression would seem nothing short of callous. Nor would anyone do this. The event precipitating the weeping is clearly negative. Moreover, the event or circumstance leading to this state is highly salient: the specific loss, bereavement, misfortune, or catastrophe will be foremost in everyone’s mind – both for the observer as well as the grieving individual. When interacting with someone experiencing stress, admitting to the stressed individual (or to ourselves) that helping is pleasurable is apt to be deemed cold-hearted, if not immoral. Nevertheless, the positive feelings are essential if an observer is to be motivated to offer assistance.

In short, we suggest that cognitive awareness of the actual stressful context has the effect of pushing the positive feelings of compassion into the mental background, rendering the positive affect less apparent. Due to emotional contagion, we tend to assume that sadness is the sole overarching emotion felt by everyone. The confusion, we propose, is understandable, but not an accurate account of the rather contrasting states felt by the observer and weeper.

If mixed emotions commonly arise in such circumstances, this raises the question of whether the positive-to-negative proportions in this mixture might differ from person to person.

## Trait Empathy

As noted earlier, people differ in whether or not they enjoy listening to nominally sad music. If compassion is a common positive emotion induced by tragic or sad displays, might listener differences in reported enjoyment be attributable to personal variability with respect to the feeling of compassion? This conjecture suggests we consider research related to trait empathy.

Empathy is commonly defined as the process by which an observer comes to understand and feel what others are experiencing. For over 200 years, scholars have suggested that there are at least two distinguishable components to empathy: the affective and cognitive elements (e.g., Smith, 1759; Spencer, 1870). The affective element consists of an involuntary emotional reaction induced in response to the observed emotions of others. The cognitive element consists of an intellectualized recognition or a simple understanding of the emotional state of others – without the observer necessarily experiencing any emotion.

In recent years, research has helped to delineate the behavioral, neuroanatomical, and neurochemical correlates for both affective empathy and cognitive empathy (Shamay-Tsoory and Aharon-Peretz, 2007; Shamay-Tsoory et al., 2009; Hurlemann et al., 2010; Lackner et al., 2010). Cognitive empathy plays an essential role in perspective-taking and is thought to provide the basis for the ability to attribute mental states (such as beliefs, intentions, and desires) to other people (Premack and Woodruff, 1978; Dennett, 1987; Baron-Cohen, 1995). The pertinent neuroanatomical network includes the ventromedial and dorsomedial prefrontal cortices, the temporoparietal junction, and the medial temporal lobe. Cognitive empathy develops comparatively late, during adolescence, and is linked to dopamine (Shamay-Tsoory, 2011).

By comparison, affective empathy includes emotion recognition, emotional contagion, motor empathy, and shared pain. The pertinent neuroanatomical network has been shown to include the inferior frontal gyrus, inferior parietal lobe, the anterior cingulate cortex, and anterior insula. Notably, the inferior frontal gyrus is part of the human mirror neuron system (Kilner et al., 2009), and is also involved in the internal simulation of emotional facial expressions (Van der Gaag et al., 2007). Affective empathy is thought to develop early in infancy, and is linked to oxytocin (Shamay-Tsoory, 2011).

People differ in the degree to which they experience empathy. First, there are developmental differences. For example, infants commonly respond to the distress of others by experiencing distress themselves. At roughly 2 years of age, toddlers commonly exhibit helping behaviors toward those in distress. Second, there are well-documented sex differences, with females self-reporting experiencing empathy more readily than males. These sex differences are less apparent in the case of psychophysiological and non-verbal behavioral measures, and most marked in the case of verbal self-report (Eisenberg and Lennon, 1983). Third, apart from age and sex differences, there are stable personality or trait differences: some people are simply more empathetic than others.

Davis (1980) conducted pivotal research investigating how people differ with respect to the experience of empathy. His research led to the development of a standard four-factor model of empathy that forms the basis for the Interpersonal Reactivity Index (IRI), a validated survey instrument for characterizing trait empathy (Davis, 1980, 1983). Davis’s research suggests that trait empathy can be characterized by four stable factors, referred to as empathic concern, personal distress, perspective taking, and fantasy. The subscales of the IRI have been associated with individual differences at the neural level, including greater gray matter volume in brain regions belonging to the mirror neuron system (Cheng et al., 2009), and greater activation of the mirror neuron system while observing facial expressions (Pfeifer et al., 2008) or listening to action sounds (Gazzola et al., 2006).

*Empathic concern* is the disposition to feel concern, sympathy, or compassion for another person experiencing some stress or misfortune. We might summarize this facet of empathy via the statement “I feel sympathy for you.” *Personal distress* is the disposition to mirror or echo feelings of personal anxiety or unease when witnessing stress or tension in others (“I feel your pain”). *Perspective taking* is the cognitive tendency to spontaneously adopt the psychological point of view of others (“I understand where you’re coming from”). Finally, *fantasy* is ostensibly the cognitive ability to be absorbed and imagine the feeling state for a fictitious character, such as portrayed in literature, drama, or film (“I can imagine that situation”). Together, empathic concern and personal distress are thought to index the affective or emotional aspect of empathy, whereas perspective taking and fantasy are thought to index the cognitive aspect of empathy.

**FIGURE 1:**
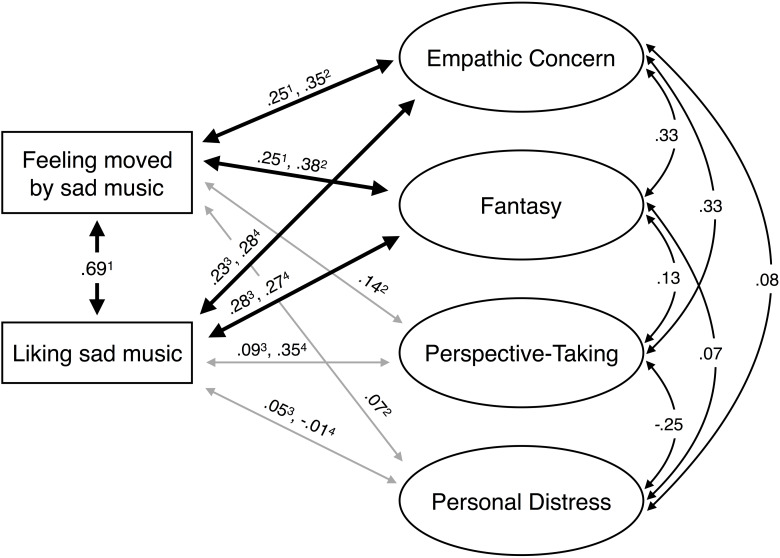
A visualization of the correlations between the subscales of the Interpersonal Reactivity Index (IRI) (a measure of trait empathy; Davis, 1980), liking for nominally sad music excerpts, and feelings of being moved evoked by nominally sad excerpts. Note that the generally low correlations are addressed in the “Discussion” section. The correlations between the trait empathy subscales (Empathic Concern, Fantasy, Perspective-Taking, and Fantasy) are taken from Davis (1983); (*N* = 1344, American undergraduates). The studies and sample sizes for the other correlations are listed below. ^1^Vuoskoski and Eerola (2017): *N* = 308, diverse nationalities. ^2^Eerola et al. (2016): *N* = 102, a representative sample of Finnish adults. ^3^Vuoskoski et al. (2012): *N* = 131, Finnish university students. ^4^Kawakami and Katahira (2015): *N* = 84, Japanese children (mean age: 11.9 years).

A number of studies have investigated the relationship between trait empathy and the enjoyment of sad music. Vuoskoski et al. (2012) exposed participants to a variety of happy, scary, tender, and sad music excerpts. Participants rated their felt emotions and preferences. In addition, participants completed the IRI. The main result of this experiment was that those participants who most enjoyed sad music scored high on empathic concern (*r* = 0.23) and fantasy (*r* = 0.28). However, in the case of personal distress and perspective taking, there was no association with a preference for sad music (*r* = 0.05 and *r* = 0.09, respectively). This same pattern of results has been replicated in multiple subsequent studies conducted in Finland (Eerola et al., 2016; Vuoskoski and Eerola, 2017), Japan (Kawakami and Katahira, 2015), and Austria (Sattmann and Parncutt, 2018). A visualization of the relations between liking and feelings of being moved evoked by nominally sad music, and subscales of trait empathy, is displayed in Figure 1.

Focusing on the affective facets of empathy, these results might be summarized as follows: Those listeners who most enjoy nominally sad music tend to experience high levels of empathic concern with nominal personal distress. Said another way, sad-music lovers experience only moderate levels of “I feel your pain” but high levels of “I feel sympathy for you.” If, as we have argued, compassion is a positively valenced affect, then sad-music lovers would appear to experience high levels of positive compassion while experiencing only nominal levels of empathetic pain. Consequently, the overall experience for these listeners would favor a positive affect.

It should be noted that all five studies cited above employed listening experiments that relied on experimenter-selected (unfamiliar) nominally sad music. It is possible that the use of unfamiliar music, or music deemed by experimenters to be “sad” is biased in some way. Two survey studies provide converging evidence that circumvent this possible confound. Garrido and Schubert (2011) and Taruffi and Koelsch (2014) conducted survey studies and found similar patterns of correlations between trait empathy and an overall preference for nominally sad music. Since these surveys allowed respondents to interpret in their own way “sad music” or “music which makes me feel sadness or grief” (Garrido and Schubert) these studies provide complementary evidence consistent with the broad picture of the association between trait empathy and sad music preferences.

Delving deeper into the principles underlying the evocation of sadness through music, Taruffi and Koelsch (2014) discovered that global trait empathy (and its subscales fantasy and empathic concern) correlated most significantly with emotion induction via *social functions* (*r* = 0.40; “Sad music makes me feel sad because I am touched by the sadness of others”), further suggesting a link between music-induced sadness and compassionate responding to others’ emotions. It should also be noted that, besides correlating with the enjoyment of sad music, trait empathy has also been associated with the enjoyment tender music (but not happy, scary, or intense music; Vuoskoski et al., 2012; Greenberg et al., 2015). We will address the overlap between compassion and tenderness later in this article (see section “Confabulation”).

## Interlude

Our theory raises at least three questions: First, if compassion is positively valenced, then wouldn’t the most enjoyable tragedies be real-world tragedies? As David Hume noted, if we enjoy sad or tragic displays, then a visit to a hospital ward should be highly pleasurable (Hume, 1759/2004, p. 243). Secondly, if compassion is a common feeling evoked for sad-music lovers, who does the listener feel compassion for? Especially in the case of purely instrumental music, it is not clear there is any sad or stressed agent who would warrant a listener’s feeling of compassion. Finally, if compassion is the predominant emotion evoked in sad-music lovers, why don’t listeners commonly describe their experience as evoking the feeling of compassion? In fact, the authors are not aware of a single study of music-induced emotion that points to compassion, sympathy, or pity as a significant reported musically evoked affect. Below we address each of these three questions in turn.

## Fiction and Fantasy

In attempting to resolve the paradox of tragedy, a number of philosophers have emphasized the fictive aspect of artistic portrayals. The idea is that because spectators recognize that the tragedy is not real, true sadness is not evoked in viewers and so audience members do not experience true negative feelings. Although the idea that factual stories have a greater capacity for evoking emotion than fictional stories is intuitively appealing, the idea is not supported by the empirical research (Strange and Leung, 1999; Green and Brock, 2000). Whether the same story is presented as fictional or factual has no effect on induced emotion. Instead, the key to induced emotion is the degree to which the spectator is transported into the portrayed world. When equally engaged in a factual or fictional narrative, the experienced emotions are equally intense (cf. Green et al., 2012; Koopman, 2015).

Recall that another way in which sad-music lovers differ from those who dislike sad music is that they tend to score high on *fantasy*. In fact, of the four factors in Davis’s model of empathy, fantasy exhibits the highest observed correlation with the enjoyment of sad music.

As noted earlier, fantasy measures the tendency for a person to become absorbed or transported into narratives such as in books or films; it reflects the degree to which a person identifies with the protagonists or characters in such narratives. In short, fantasy indexes that facet of personality most likely to amplify the affective experience. Fantasy is predictive of strong emotional reactions not just in narrative or literary contexts, but, as we have just noted, in musical contexts as well (e.g., Harris et al., 2000; Vuoskoski et al., 2012; Eerola et al., 2016). Although fantasy is considered to be a form of cognitive rather than affective empathy, it is possible that fantasy has a catalyzing or amplifying effect – causing listeners to experience greater compassion. It may be that music-induced compassion is particularly reliant on imagination and the tendency to identify with objects of imagination (cf. the Fantasy-subscale of trait empathy; Davis, 1980).

Whether a portrayal is fictional or factual, recognizing the artificial nature of the art or entertainment itself remains important because spectators are absolved of the need to offer altruistic assistance. When watching Shakespeare’s *Romeo and Juliet*, audience members do not feel compelled to rush the stage and snatch the dagger from Juliet’s trembling hand. Nor do we feel the urge to toss food at the starving cast members of Victor Hugo’s *Les Misérables*. In this regard, portrayals of tragedies (either fictional or documentary) have an advantage over actual (real, unfolding) tragedies, since viewers can experience the positive feelings of compassion without feeling any guilt for failing to engage in compassionate acts. Nor is there any cost to the observer, such as the need to share resources with a stressed protagonist or the erosion of social reputation. As audience members, we sit in silence, entirely untroubled by our failure to help. We feel pity or sympathy without being burdened by the inconvenience of intervening or the guilt of inaction. In short, Hume’s critique does not apply because, in contrast to actual tragedies, the compassion evoked in artistic or entertainment contexts is cost-free. The spectator does not have to part with precious resources, worry about his/her reputation, fear social ostracism for failing to come to the assistance of others, or wrestle with feelings of guilt.

In addressing Hume’s objection, a further consideration is the magnitude and vividness of the portrayed tragedy or stressor. Graphic portrayals of pain or torture may evoke considerably more personal distress compared with portrayals of grief or sadness. Hence, a person who finds artistic representations of sadness enjoyable may nevertheless find representations of more stressful scenarios distressing and unpleasant.

In this regard, we might also note that different circumstances may not always produce the same balance between empathetic concern and personal distress. A visit to a hospital ward might indeed evoke more compassion, but we would also expect a hospital visit to evoke more personal distress. These increases may or may not be equivalent or proportional. Indeed, compared with a tragic play or film, a visit to a hospital ward may induce much more personal distress while only moderately increasing the feeling of compassion.

Finally, we might note that artistic portrayals of tragedy (both fictional and documentary) offer contexts that allow spectators to more readily recognize or acknowledge the positive state induced by compassionate feelings. Leaving a theater after watching a tragic drama, it would be socially acceptable to declare feelings of enjoyment. Leaving a funeral parlor after comforting bereaved friends, it would be socially unacceptable and personally disturbing to acknowledging such positive feelings even if they are present.

## Compassion for Whom?

If nominally sad music is able to evoke compassion, an obvious question is “compassion for whom?” In literature and drama, most portrayals of human characters are recognizable and salient; it makes sense that spectators might empathize with various characters. In the case of vocal music, sad lyrics may make it possible to empathize with the singer or the character conveyed in the lyrics. However, listeners have long reported that sadness can be compellingly conveyed (and enjoyed) by purely instrumental music. It is sad instrumental music that most clearly raises the conundrum of agency: who is the object of our purported feeling of compassion? Do listeners feel compassion for the oboe or the electric guitar? Do we imagine that the instrumentalist is in a state of grief? Do we somehow feel pity for the absent composer?

Philosopher Stephen Davies has been explicit in articulating these misgivings. Davies argues that music is not an agent, and so by definition, music cannot be sad (Davies, 1997b). If Davies is right, then the corollary would be that music cannot be the target of listener compassion. When viewed from a logical perspective, we ought not to feel any socially oriented emotion toward an entity that is not an agent capable of feeling pain or distress. However, these arguments presume that humans are strictly rational beings, and that human emotions are driven predominantly or solely by cognitive/logical assessments. Research in the fields of affective neuroscience, behavioral economics, and ethology suggest this assumption is untenable (e.g., Tversky and Kahneman, 1986; Damasio, 1994; Kahneman, 2011). Similar agency-related paradoxes are evident in other domains, such as pornography where printed photographs or illuminated computer screens can cause sexual arousal in observers, despite the complete absence of any human presence that would warrant feelings or behaviors whose ultimate purpose is pair-bonding or procreation. More generally, humans have been shown to have an automatic tendency to “mind-read” non-human and virtual objects, such as moving geometric figures (Heider and Simmel, 1944) – and assign intentions to them. Empirical research has shown this to be the case with music as well, demonstrating that listeners do in fact have a propensity to hear music as if it were mimicking or representing the actions and intentions of a (virtual) person (e.g., Watt and Ash, 1998; Aucouturier and Canonne, 2017).

We propose that the resolution to the problem of agency (offered in the ensuing section) will also address our third question: Why don’t sad-music lovers commonly describe their listening experience as evoking a feeling of compassion?

## Confabulation

As already noted, the authors are not aware of any study of music-induced emotion that points to compassion, sympathy, or pity as a significant reported musically evoked affect. If, as we have argued, compassion is the principal induced affect for sad-music lovers, why aren’t feelings of compassion commonly reported when listening to nominally sad music?

Zentner et al. (2008) have identified the most common descriptions listeners use to characterize various music-induced emotions. Although it is common for listeners to report feelings of sadness, common descriptions also include feelings of *being touched* and feelings of *tenderness* (*attendri* in French; also translatable as “mellowed” or “softened up”).

Supplementing this research, recent scholarship on arts-related emotions has pointed to the concept of *being moved* (Konečni, 2005, 2015; Bråten, 2007; Cova and Deonna, 2014; Hanich et al., 2014; Kuehnast et al., 2014; Menninghaus et al., 2015; Schubert and Seibt, 2017). “Being moved” has figured prominently in theoretical and empirical work conducted by Menninghaus and his colleagues. They conceptualize “being moved” as a mixed but predominantly positive emotion that plays an important role in the enjoyment of tragic art (Hanich et al., 2014; Kuehnast et al., 2014; Menninghaus et al., 2015). Importantly, Menninghaus et al. (2015) have emphasized the empirical evidence linking “being moved” with prosocial/social bonding behaviors. At the same time, they suggest that this prosocial function is largely opaque to spectators. With regard to art-elicited instances of “being moved” and prosocial motivations they write:

“…classical treatises on being moved by art barely ever explicitly speak of such (prosocial) norms. In fact, art-works that explicitly propagate such prosocial norms and self-ideals are often, if not mostly, bad art. We therefore suggest that it may be important for the poetics of being moved that the prosocial implications of this feeling largely escape a conscious representation and are only brought to the fore by scientific analysis.” (Menninghaus et al., 2015; p. 24).

If both being moved and compassion are predominantly positive feeling states that encourage prosocial behavior, is it possible that they may be one-and-the-same emotion? The research suggests a high degree of overlap: Being moved has been conceptualized as the common vernacular of a broader social-relational emotion encompassing feelings of compassion, tenderness, patriotism, and other related experiences (Fiske et al., 2019), and trait empathic concern (i.e., the tendency to experience compassion; Davis, 1980) consistently predicts self-reported feelings of being moved (Eerola et al., 2016; Vuoskoski and Eerola, 2017; Zickfeld et al., 2017).

In the case of instrumental music, the absence of an obvious stressed agent might well be expected to discourage listeners from describing their feeling as one of compassion, given that feeling compassion implicitly entails the desire to help and care for some agent in need. Accordingly, common descriptions in response to sad music, like “feeling tender,” “being touched,” “being moved,” or “tugging on one’s heartstrings,” appear to be useful synonyms, and more fitting for the “floating intentionality” of music (cf. Cross, 2005). Furthermore, if one of the purposes of weeping as an ethological signal is to terminate aggressive behaviors by an observer, then other common sad-music-induced descriptions such as “feeling peaceful” or a feeling of “gentleness” are precisely what one would predict. In contrast to the word “compassion,” these terms may be preferred because they circumvent the awkward implication that there must be a target agent in need. These examples notwithstanding, it is also not unknown for sad-music lovers to characterize their feelings using terms like “kindness,” “affection,” and “devotion”—descriptors that are more explicitly suggestive of a target recipient.

We concur with Menninghaus and colleagues who have suggested that the prosocial character of the induced emotions largely escapes conscious awareness. We would add to this observation that even when listeners recognize feelings of sympathy, pity, or compassion, they tend to reject such prosocial interpretations as inappropriate in the case of music. In short, we propose that listeners tend to confabulate their experiences, offering a spurious rationale that avoids the perplexing implication that music is a person or conscious agent.

Curiously, although listeners may be reluctant to describe their emotional responses to music using prosocial language, listeners have long been comfortable describing musical passages as representing the behaviors of virtual actors. Composer Robert Schumann, for example, famously described works and passages in the music of Frédéric Chopin by writing detailed narratives involving imagined characters. In modern times, such musical descriptions involving virtual actors have been regarded simply as romantic flights of creative imagination. However, recent music analytic scholarship has drawn attention to the ubiquity of virtual agency, including movement, gesture, embodiment, and social narrative as a foundation for music-induced emotion (cf., Hatten, 2018).

This still leaves the question of the feeling of *sadness*. Recall that the most common term listeners use to describe their feelings when listening to nominally sad music is that it simply makes them feel *sad*. Although not universal, this description is common, both among sad-music lovers, and those listeners who dislike sad music.

In order to better explore the relationship between sadness and enjoyment, Vuoskoski and Eerola (2017) carried out an experiment that specifically explored the pleasure evoked by sad music. Key to this study was a mediation analysis that endeavored to trace the causal connections between *sadness*, *being moved*, and *enjoyment*. The analysis showed that felt sadness contributed to the enjoyment of sadness-inducing music by intensifying feelings of being moved. That is to say, “being moved” was pivotal in order for listeners to enjoy nominally sad music. If “being moved” and “compassion” are synonymous with an evoked prosocial disposition, then the mediation analysis can be interpreted as indicating that the causal train is not *sadness evokes pleasure*, but *sadness evokes prosocial feelings, which in turn evokes pleasure*. Notice that this latter causal scenario is consistent with an interpretation in which the feeling of sadness represents a *contagious* emotion whose purpose is to facilitate emotion recognition, and that recognizing this emotion induces the appropriate *repercussive* emotion (being moved or compassion) which is positively valenced. In short, it appears that sad-music listeners commonly experience a state of mixed emotions involving both sadness and being-moved or compassion. We would suggest that whether a listener likes or dislikes sad music, the experience of sadness is the more cognitively salient of the two affects and so dominates introspective reports.

In general, it bears noting that people have trouble describing precisely what they feel when listening to music. Moreover, the problem is compounded by the often rapid changes characteristic of music; the mood or tenor of many musical passages can change several times within a few seconds (Huron, 2006). Further research is clearly warranted in order to better understand how introspective reports relate to the presumed underlying affective experience.

## Openness to Experience?

One final topic remains to be addressed. So far, we have emphasized the relationship between sad-music enjoyment and trait empathy. For readers familiar with the research literature, it will seem unorthodox that we have ignored the most common finding linking strong experiences evoked by music and personality. Specifically, a number of studies have found that strong emotional experiences such as chills (frisson) or weeping are more common among listeners who score high on openness to experience (Rentfrow and Gosling, 2003; McCrae, 2007; Nusbaum and Silvia, 2011; Silvia and Nusbaum, 2011; Maruskin et al., 2012; Dobrota and Ercegovac, 2015; Silvia et al., 2015; Colver and El-Alayli, 2016).

However, this relationship is confounded by research showing a strong correlation between openness to experience and measures of empathy: people who score high on measures of empathy also tend to show greater openness to experience (Costa et al., 2014; Melchers et al., 2016; Sattmann and Parncutt, 2018). Following up on this observation, Sattmann and Parncutt conducted a study involving some 300 participants. They found that when a measure of empathy is included, openness to experience is no longer a significant predictor of strong emotions evoked by music. That is, they confirmed that the main personality trait predicting music-induced emotion is empathy, not openness to experience (Sattmann and Parncutt, 2018).

## Conclusion

In this article we have presented a theory whose first aim is to explain the enjoyment of nominally sad or tragic music. Notably, our theory proposes an explanation for individual differences in liking for sad music, which we regard as an essential test of any theory of sad music enjoyment. In formulating our theory, we have aimed to create an account that is plausible at multiple descriptive levels, including ethological, evolutionary, and neuroanatomical viewpoints, as well as behavioral and phenomenological perspectives.

In the restricted case of nominally sad music, *pleasurable compassion theory* posits the following logic: Nominally sad music has been shown to mimic acoustical features of melancholic and grief-related vocalizations. That is, research suggests that the “sadness” of sad music arises because the music emulates sad vocal prosody. Despite the absence of a clear agent who might be experiencing distress, empathetic listeners exposed to such music can experience both emotional contagion (evoking feelings of sadness or commiseration) as well as repercussive emotions (evoking feelings of sympathy or compassion). Commiseration and compassion are negatively and positively valenced affects, respectively. When listening to nominally sad music, the experience of those listeners who exhibit high trait empathy for *personal distress* tends to be dominated by feelings of sadness or commiseration; consequently, they tend to find the listening experience unpleasant. By contrast, the experience of those listeners who exhibit high trait *empathic concern* tends to be dominated by feelings of sympathy or compassion, and so they tend to find the listening experience enjoyable. Trait empathy for *fantasy* appears to amplify the enjoyment by increasing the listener’s absorption or engagement with the portrayed world.

Especially in the case of instrumental music, the absence of an obvious stressed character or agent discourages listeners from describing their feeling using socially loaded terms such as pity or sympathy. Consequently, synonyms like “feeling tender,” “being touched” or “being moved” are common characterizations. Self-report descriptions are broadly consistent with induced prosocial feelings – either feelings associated with terminating aggression (e.g., feeling peaceful, gentleness), or feelings associated with altruistic motivations (e.g., kindness, affection, devotion, tugging on one’s heartstrings, etc.).

More broadly, pleasurable compassion theory might be usefully applied beyond the phenomenon of sad music to tragic arts and entertainments generally. In this broader context, we might summarize the main elements of the theory as follows:

• Displays of sadness or grief can evoke both contagious and repercussive emotions in observers. A contagious echoing of sadness may be beneficial in several ways – for example, by helping an observer recognize a displayed emotion.
• Grief expressions exhibit the hallmarks of a classic ethological signal. Grief displays induce a biologically prepared disposition for observers to terminate aggression and/or offer assistance. Altruistic acts commonly offer distal or ultimate benefits to the observer through kin- or reciprocal altruism, enhanced social status for the observer with respect to the stressed individual, reputation enhancement or maintenance, or through the avoidance of social ostracism. However, with the possible exception of fear of social ostracism, these ultimate motivations are not cognitively salient. Instead, the immediate motivation to engage in altruistic acts is the proximal feeling of pity or *compassion* and the anticipated future feeling of *virtue*.
• Both altruistic acts and altruistic thoughts have been experimentally shown to induce positive emotions including the activation of brain regions associated with pleasure. Since compassion is intended to motivate altruistic acts, the feeling of compassion alone is also almost certainly a positively valenced feeling state.
• People differ in the degree to which they experience emotional contagion and repercussive emotions. Those spectators whose experience is dominated by compassion (“empathic concern” in Davis’s IRI model) experience a net positive feeling state. Conversely, those spectators whose experience is dominated by emotional contagion or commiseration (“personal distress” in the IRI model) experience a net negative feeling state.
• In real-world tragic situations observers tend to focus cognitively on the stressful event or circumstance leading to the grief expression. The specific loss, bereavement, misfortune, or catastrophe will be foremost in the observer’s mind. Consequently, the pleasure of compassion is typically subliminal, unconscious, and cognitively opaque. Observers of tragedies have little awareness of the underlying positive affect that, in real-world situations, would propel them to altruistic action.
• Artistic representations or portrayals of tragedy are commonly able to evoke biologically prepared compassionate responses. At the same time, representations or portrayals of stressful situations liberate the observer from the obligation to engage in altruistic assistance, and absolve the observer from feeling guilty or worrying about ostracism for inactivity. Consequently, fictive or documentary representations of tragedies typically hold a greater capacity for enjoyment than real-world tragedies.

Notice that our proposal for resolving the paradox of the enjoyment of tragic portrayals in the arts and entertainment avoids the traditional question: “How can feeling sad be enjoyable?” Instead, our proposal focuses on the question “Under what circumstances can the pleasure of prosocial compassion outweigh the displeasure of contagious sadness?”

## Discussion

It should be noted that pleasurable compassion theory is a hedonic theory rather than an aesthetic theory. That is, our account of the enjoyment of nominally sad music says nothing about aesthetic value. Arts or entertainments can be valued because they educate us, inspire us, challenge us, disturb us, or even insult us. The value of music (and art generally) can be entirely independent of its capacity to evoke pleasure.

A further limitation is that the theory of pleasurable compassion presented here relates to only one component of the general problem of negative emotions in the arts – namely, portrayals of sadness, grief, or tragedy. Pleasurable compassion theory does not address other negative emotions commonly associated with art or entertainment – such as aggression, fear, horror, or disgust.

Even as a hedonic theory, our theory does not preclude other possible concurrent sources of enjoyment when a spectator is exposed to stressful portrayals (including exposure to nominally sad instrumental music). Other possible sources of pleasures might include the satisfying of curiosity, positive feelings of righteous indignation, admiration for moral behavior, appreciation of the quality of artistic craft or performative skill, arbitrary learned positive associations, processing fluency (via familiarity), or other conjectured sources of enjoyment.

Although the pattern of empathetic traits associated with enjoyment of sad music has been replicated in several studies, it should be noted that the overall correlations for fantasy, compassion, and commiseration are modest (in the vicinity of *r* = 0.25). Relative low correlations may arise for several reasons, including limitations of survey instruments for measuring trait empathy, questionable choices of nominally sad musical stimuli, individual differences in musical taste or familiarity (including genre familiarity), and/or low reliability in participants’ ability to self-report.

A major limitation of the theory is that it draws almost exclusively on studies of sad music. This highlights the need for parallel studies in other arts and entertainments, notably, literature, poetry, drama, and film.

Our proposal offers a number of benefits and avoids two classic problems:

1. Pleasurable compassion theory escapes the problem of “transformation” where induced (contagious) feelings of sadness must somehow be recast or transmuted into a positive affect.
2. The theory is consistent with ethological signaling theory which explains the largely involuntary and automatic nature of observer responses to displays of stress.
3. The theory is consistent with an evolutionarily plausible account of why the feeling of compassion would exist, and why compassion would be evoked when observing stress-inducing circumstances.
4. The theory accounts for why observers would be affected by purely fictional representations. At the same time, the theory escapes a crucial criticism that plagues most theories positing positive responses to fictional tragedies—namely, that the same account would suggest that observing real-world tragedies should be even more enjoyable.
5. Most importantly, our theory accounts for empirically observed individual differences. That is, the theory is able to account for why some spectators enjoy tragic portrayals while others fail to derive any enjoyment.

As with any theory, it behooves us to consider potential problems and identify possible tests that might challenge or falsify the pleasurable compassion theory.

First, we have explicitly proposed that our theory plausibly extends beyond the experience of sad music to account for spectator emotions when exposed to other sad or tragic arts or entertainments. Since our theoretical account hinges on a personal trait factor, it would be appropriate to test whether people who enjoy listening to nominally sad music also tend to enjoy tragic drama, film, or literature compared with other people. Moreover, it would be appropriate to test whether spectators who enjoy such non-musical tragedies also tend to score high on empathic concern and fantasy according to the IRI.

With regard to physiological and behavioral effects, research pertaining to the effects of oxytocin suggests that empathy and prosocial behaviors are complex subjects with many competing interpretations of past endocrine findings (e.g., Kemp and Guastella, 2010). Nevertheless, focusing on behavioral effects alone, one possible avenue of research might test the prediction that those individuals who most enjoy nominally sad music would be more likely to exhibit trusting behaviors following sad music exposure (Theodoridou et al., 2009).

A possible weakness of our theory is explaining the role of empathetic fantasy. Recall that fantasy exhibits the highest correlation with the enjoyment of nominally sad music. Fantasy is usually described as the cognitive ability to project oneself into a scenario, such as imagining the feeling state of a fictional character. Although this concept makes sense for narrative arts like literature or drama, it seems less fitting in the case of music. Do high-fantasy listeners “identify” more with a violin protagonist? Do they better imagine themselves in a fictional acoustical world? It is certainly plausible that listeners with high fantasy-proneness may be more likely to experience music as conveying a strong sense of agency, whether real or imagined. Nevertheless, what does it mean for a listener to be more engaged (high fantasy) with musical sounds?

Two possible sources of fantasy are music-induced visual imagery and music-induced mind-wandering. Listeners sometimes experience visual imagery when listening to music (Halpern, 1992). Such music-induced visual imagery can influence listener emotion. For example, (Baltes and Miu (2014), p. 62) found modest evidence suggesting that visual imagery facilitates music-induced chills. More commonly, music listening is associated with various forms of mind-wandering. Taruffi et al. (2017) found that nominally sad music was significantly more likely (than happy music, controlled for tempo) to evoke mind-wandering in listeners. Furthermore, they observed a similar pattern in the activity of the Default Mode Network (fMRI, *N* = 24): sad music was associated with greater centrality of the nodes of the DMN. In general, the default mode network is thought to play an important role in simulation, including simulation of other minds and social content. Tamir et al. (2015) have observed precisely this DMN/simulation relationship in the context of reading fiction, as well as an association between the degree of DMN activation and empathic ability (Tamir et al., 2015). In the case of music, a possible test of this “mind-wandering” conjecture might investigate whether those individuals exhibiting high fantasy are more prone to experiencing greater mind-wandering during sad music listening. Perhaps sad-music enjoyment is linked with a specific type of mind-wandering.

A related outstanding issue is clarifying the relationship between fantasy and empathic concern. It turns out that IRI fantasy scores alone are predictive of sad-music enjoyment independent of empathic concern. If enhanced empathetic concern is not essential for sad-music enjoyment, then the theory of pleasurable compassion presented in this article is immediately thrown into doubt. However, a detailed examination of the fantasy items in the IRI raises doubts about the independence of this facet. Critically, the fantasy items in the IRI imply that becoming absorbed or immersed in a book or film is inherently enjoyable. For example, IRI test items include arguably biased phrases such as “an *interesting* story” and “a *good* film,” making it less likely that people who score high on the fantasy scale would commonly experience negative or aversive emotions in fictional or artistic contexts. In other words, the fantasy scale already appears to tap into predominantly positive experiences of immersion and/or identification. This apparent confounding effect is echoed in a moderate positive correlation between empathic concern and fantasy (in the region of *r* = 0.30–0.36 according to Davis, 1980, 1983).

This is not to claim that fantasy or absorption is not important. “Getting lost” in a work enhances its emotional impact. In their work on “transportation,” Green et al. (2004) observe that “A common complaint from individuals who have watched a bad movie or read a dull novel is that they “just couldn’t get into it.” (p. 314). Our claim is simply that in order for tragedy-induced empathy to be experienced as pleasurable, the principal evoked empathetic response needs to be prosocial empathic concern rather than personal distress.

The value of pleasurable compassion theory remains to be seen. However, we hope that the theory provides a useful framework that might inform some aspects of future research pertaining to the paradox of enjoying tragic arts and entertainments.

## Author Contributions

DH conceived the main idea. DH and JV contributed to developing the theory and writing the manuscript.

## Conflict of Interest

The authors declare that the research was conducted in the absence of any commercial or financial relationships that could be construed as a potential conflict of interest.
